# ‘Smoking Genes’: A Genetic Association Study

**DOI:** 10.1371/journal.pone.0026668

**Published:** 2011-10-26

**Authors:** Zoraida Verde, Catalina Santiago, José Miguel Rodríguez González-Moro, Pilar de Lucas Ramos, Soledad López Martín, Fernando Bandrés, Alejandro Lucia, Félix Gómez-Gallego

**Affiliations:** 1 Department of Biomedicine, Universidad Europea de Madrid, Villaviciosa de Odón, Comunidad de Madrid, Spain; 2 Department of Neumology, Hospital Gregorio Marañón, Madrid, Comunidad de Madrid, Spain; 3 Department of ‘Aula de Estudios Avanzados’, Fundación Tejerina, Madrid, Comunidad de Madrid, Spain; University Hospital Vall d'Hebron, Spain

## Abstract

Some controversy exists on the specific genetic variants that are associated with nicotine dependence and smoking-related phenotypes. The purpose of this study was to analyse the association of smoking status and smoking-related phenotypes (included nicotine dependence) with 17 candidate genetic variants: CYP2A6*1×2, CYP2A6*2 (1799T>A) [rs1801272], CYP2A6*9 (−48T>G) [rs28399433], CYP2A6*12, CYP2A13*2 *(*3375C>T) [rs8192789], CYP2A13*3 (7520C>G), CYP2A13*4 (579G>A), CYP2A13*7 (578C>T) [rs72552266], CYP2B6*4 (785A>G), CYP2B6*9 (516G>T), *CHRNA3* 546C>T [rs578776], *CHRNA5* 1192G>A [rs16969968], *CNR1* 3764C>G [rs6928499], *DRD2-ANKK1* 2137G>A (*Taq1A*) [rs1800497], *5HTT* LPR, *HTR2A* −1438A>G [rs6311] and *OPRM1* 118A>G [rs1799971]. We studied the genotypes of the aforementioned polymorphisms in a cohort of Spanish smokers (*cases*, N = 126) and ethnically matched never smokers (*controls*, N = 80). The results showed significant between-group differences for CYP2A6*2 and CYP2A6*12 (both *P*<0.001). Compared with carriers of variant alleles, the odds ratio (OR) for being a non-smoker in individuals with the wild-type genotype of CYP2A6*12 and *DRD2-ANKK1* 2137G>A (*Taq1A*) polymorphisms was 3.60 (95%CI: 1.75, 7.44) and 2.63 (95%CI: 1.41, 4.89) respectively. Compared with the wild-type genotype, the OR for being a non-smoker in carriers of the minor CYP2A6*2 allele was 1.80 (95%CI: 1.24, 2.65). We found a significant genotype effect (all *P*≤0.017) for the following smoking-related phenotypes: (i) cigarettes smoked per day and CYP2A13*3; (ii) pack years smoked and CYP2A6*2, CYP2A6*1×2, CYP2A13*7, CYP2B6*4 and *DRD2-ANKK1* 2137G>A (*Taq1A*); (iii) nicotine dependence (assessed with the Fagestrom test) and *CYP2A*6*9. Overall, our results suggest that genetic variants potentially involved in nicotine metabolization (mainly, *CYP2A*6 polymorphisms) are those showing the strongest association with smoking-related phenotypes, as opposed to genetic variants influencing the brain effects of nicotine, e.g., through nicotinic acetylcholine (*CHRNA5)*, serotoninergic (*HTR2A*), opioid (*OPRM1*) or cannabinoid receptors (*CNR1*).

## Introduction

Cigarette smoking is the single most preventable cause of lung cancer and a main source of morbimortality worldwide [1]. Smoking quit rates are low (∼10% after 6 months) [2] and do not increase substantially with pharmacological treatment [2], [3]. Further, long-term (i.e. years) abstinence following treatment is rare. Nicotine dependence is a main factor contributing to maintaining the harmful cigarette smoking behavior [4], [5]. Thus, to indentify the main causes of nicotine dependence and smoking-related phenotypes is of medical interest.

Evidence from classic studies on twins [6]-[10] and more recent molecular approaches including wide genome linkage studies [11]–[17] indicate that smoking-related phenotypes, particularly nicotine dependence are highly heritable (for a review, see [18], [19]). More controversy exists on the specific genetic variants that have a functional significance on such phenotypes, with a strong rationale existing for polymorphisms in genes encoding nicotine-metabolizing enzymes in the liver [cytochrome P450 2A6 (*CYP2A6*) and B6 (*CYP2B6*)] and lungs (*CYP2A13*) [18]. Other candidate polymorphisms are in genes encoding neuronal nicotinic acetylcholine receptors (*CHRNA3 and CHRN5*), or in genes involved in dopaminergic, serotoninergic, cannabinoid and opioid pathways related to nicotine reward and dependence, such as dopamine D_2_ receptor/ankyrin repeat and kinase domain containing 1 (*DRD2/ANKK1* dopamine D2), serotoninergic transporter [*5-HTT*, also termed solute carrier family 6, member 4 (*SLC6A4*)] and receptor (*HTR2A*), cannabinoid receptor 1 (*CNR1*) and mu opioid receptor (*OPRM1*) [18].

The purpose of this study was to assess the association of smoking status and smoking-related phenotypes (included nicotine dependence) with 17 candidate genetic variants: CYP2A6*1×2, CYP2A6*2 (1799T>A) [rs1801272], CYP2A6*9 (-48T>G) [rs28399433], CYP2A6*12, CYP2A13*2 *(*3375C>T) [rs8192789], CYP2A13*3 (7520C>G), CYP2A13*4 (579G>A), CYP2A13*7 (578C>T) [rs72552266] , CYP2B6*4 (785A>G), CYP2B6*9 (516G>T), *CHRNA3* 546C>T [rs578776], *CHRNA5* 1192G>A [rs16969968], *CNR1* 3764C>G [rs6928499], *DRD2-ANKK1* 2137G>A (*Taq1A*) [rs1800497], *5HTT* LPR, *HTR2A* -1438A>G [rs6311] and *OPRM1* 118A>G [rs1799971]. We studied the genotypes of the aforementioned polymorphisms in a cohort of Spanish smokers (*cases*) and ethnically-matched non-smokers (*controls*).

## Materials and Methods

### Participants

Written consent was obtained from each participant. The study protocol was approved by the institutional ethics committee (*Universidad Europea de Madrid* (UEM). Spain) and was in accordance with the Declaration of Helsinki for Human Research of 1974 (last modified in 2000).

A total of 206 individuals [all unrelated to each other and of the same Caucasian (Spanish) descent for 3 or more generations] enrolled in the study, including 126 smokers (*cases,* 64 male 62 female, mean age 54±14 years, range 20–84) and 80 never-smokers (*controls,* 37 male 43 female, mean age 42±11 years, range 24–66). In the smokers' group, 56 people were unhealthy smokers (diagnosed with lung cancer) and 70 were healthy at the time of the study. All cases met the following three criteria: 1) smoked more tan 10 cigarettes per day at the time of the study, 2) had a smoking history of more than 10 packs per year and 3) had more than 3 scores in the Fagerstrom Test for Nicotine Dependence (see below). Participants in the control group were life-time never smokers who had taken at least one puff from a cigarette in their life-time without developing a pattern of regular smoking.

### Phenotype assessment

Nicotine dependence was assessed with the Fagerstrom Test for Nicotine Dependence (FTND) [20]. The FTND is a six-item questionnaire (score range 0–10) that is widely used to evaluate the severity of nicotine dependence. Regular smokers were divided in low-dependence (0–3 scores), medium-dependence (4–6 scores) and high-dependence smokers (7–10 scores) according to this scale.

Exposure to tobacco smoke (tobacco consumption) was assessed as self-reported cigarettes per day (CPD) in the last year and pack years smoked (PYS). The PYS is used to describe the number of cigarettes a person has smoked over a lifetime, e.g. 1 PYS is defined as 20 manufactured cigarettes (one pack) smoked per day for one year.

### Genotype assessment

During 2006–2009, we extracted blood leukocyte DNA from the participants using a standard phenol chloroform protocol and performed genotype analyses in the genetics laboratory of the *Universidad Europea de Madrid* (Spain). Our study followed recent recommendations for replicating genotype-phenotype association studies [21]: genotyping was performed specifically for research purposes, and the researchers in charge of genotyping were totally blinded to the participants' identities (blood samples were tracked solely with bar-coding and personal identities were only made available to the main study researcher who was not involved in actual genotyping).

All genotyping was conducted by polymerase chain reaction (PCR). Allele-specific PCR methods were applied for the detection of *5-HTT* LPR. The PCR products were then analyzed directly by 1.2% agarose gel electrophoresis. Genotyping of CYP2A6*12 and CYP2A6*1×2 was performed by a nested PCR method according to previously described protocols [22], [23]. The genotypes of CYP2A13*2 [rs8192789], CYP2A13*3, *CHRNA5* 1192G>A [rs16969968] and *HTR2A* -1438A>G [rs6311] were analyzed by PCR followed by Restriction Fragment Length Polymorphisms (RFLPs); the PCR products were digested with *HhaI*, *MspI, Taq^α^I* and *MspI* respectively (New England Biolabs, Inc., Beverly, MA). For all PCR-RFLP assays, the digested amplicons were separated on a 1.5% agarose ethidium bromide-stained gel. Genotyping of CYP2A6*2 [rs1801272], CYP2A6*9 [rs28399433], *CYP2A13* 579G>A, *CYP2A13* 578C>T [rs72552266], CYP2B6*4 785A>G, CYP2B6*9 516G>T and *CHRNA3* 546C>T [rs578776] were performed with the single-base extension (SBE) system (ABI Prism SNaPshot Multiplex Kit, Applied Biosystems, Foster City, CA).

For *OPRM1* 118A>G [rs1799971] and *DRD2-ANKK1* 2137G>A (*Taq1A*) [rs1800497] genotyping we used real-time PCR followed by melting curve analysis with fluorescence resonance energy transfer (FRET) probes with a thermal cycler (Light Cycler 2.0 IVD, Roche Diagnostics, Barcelona, Spain). Real-time PCR and Taqman probes were used to asses *CNR1* 3764C>G [rs6928499] with a Step One Real-Time PCR System (Applied Biosystems, Foster City, CA).

### Statistical analysis

The chi-squared (χ2) test was used to assess deviations of genotype distribution from the Hardy-Weinberg equilibrium (HWE) in the whole study sample (cases+controls), and in the control group. We also compared mean values of smoking phenotypes (years smoking, CPD, FTND, PYS) between genders using the Student's unpaired *t* test. The level of significance was set at 0.05 for the two aforementioned analyses.

To compare smokers vs. non-smokers (*case:control study*), we used: (i) the χ^2^ test for between-group comparisons of genotype frequencies, and (ii) logistic regression to calculate the odds ratio (OR) of being a non-smoker based on the studied polymorphisms. Between-group comparisons of genotype frequencies were corrected for multiple comparisons using the Bonferroni method, in which the threshold *P*-value is obtained by dividing 0.05 by the number of comparisons, i.e. n = 17, corresponding to the 17 polymorphisms we studied (thus, threshold *P*-value = 0.003).

To assess genotype associations with smoking-related phenotypes within the smokers' group (*cohort study*), we used the ANOVA test to compare mean values of nicotine dependence (assessed with the FTND), CPD and PYS among the different genotypes of each polymorphism. The threshold *P-*value was obtained by dividing 0.05 by the number of comparisons for each polymorphism, i.e. n = 3, corresponding to each genotype (thus, threshold *P-*value = 0.017).

All statistical analyses were performed with the PASW/SPSS Statistics 18.0 (SPSS Inc, Chicago, IL).

## Results

### Smoking phenotypes in cases (smokers)

The main values of smoking-related phenotypes in the smokers' group are shown in Table 1. Participants in this group showed a strong nicotine dependence and high levels of tobacco consumption; 66% of the total group were heavy smokers (CPD≥1 pack/day) and 60% had medium-high nicotine dependence (≥4 scores in the FTND). Women (46±11 years) tended to be younger than men (61±12 years) (*P* = 0.129); as such, they had been smoking for fewer years, and had lower values of CPD and PYS than men (all *P*<0.001). The FTND score was similar in both genders (*P* = 0.48).

**Table 1:** Main characteristics of the smokers' group.

Smoking phenotypes	Men	Women	Total (men+women)	*P* for between-gender comparison
Years smoking	40.2±12.9	27.1± 8.4	33.7±12.7	**<0.001**
CPD	30.7±10.4	21.8± 8.1	26.5±10.0	**<0.001**
PYS	53.0±26.2	26.4±10.3	39.9±24.0	**<0.001**
FTND	5.7±2.2	6.0±2.0	5.8±2.1	0.48

Abbreviations: CPD, cigarettes per day; FTND, Fargestrom Test for Nicotine Dependence; PYS, pack years smoked (describes the number of cigarettes a person has smoked over long periods of time, e.g. 1 PYS = 20 cigarettes (one pack) smoked per day for one year). Significant *P-*values for between-gender comparisons are shown in bold.

Data are mean±SD.

### Case-control study: Genotype comparisons between the two study groups

Genotype success in the whole study sample was 99.88%, with no failures observed in the smokers' group. All genotype distributions were in HWE in the whole study sample (cases+controls) except for CYP2A6*2 (*P* = 0.001), *CHRNA3* 546C>T (*P* = 0.013), *OPRM1* 118A>G (*P* = 0.02) and *DRD2-ANKK1* 2137G>A (*Taq1A*) (*P* = 0.02). In the control group, all genotype distributions were in HWE except for CYP2A6*2 (*P* = 0.02), *5-HTT* LPR (*P* = 0.007) and *DRD2-ANKK1* 2137G>A (*Taq1A*) (*P* = 0.02).

No between-gender differences were found for the whole study sample, except for CYP2A6*1×2 and *DRD2-ANKK1* 2137G>A (data not shown).

Genotype frequency distributions in the two study groups are shown in Table 2. We found *P-*values below 0.05 for between-group comparisons in CYP2A6*2, CYP2A6*12, CYP2A6*1x2, CYP2A13*2, *CHRNA3* 546C>T, *DRD2-ANKK1* 2137G>A (*Taq1A*) and *5-HTT* LPR; yet, after adjustment for multiple comparisons statistical significance remained only for CYP2A6*2 and CYP2A6*12 (both *P*<0.001, and thus below the threshold *P-*value of 0.003). Compared with carriers of variant alleles, the OR for being a non-smoker in individuals with the wild-type genotype of CYP2A6*12 and *DRD2-ANKK1* 2137G>A (*Taq1A*) polymorphisms was 3.60 (95%CI: 1.75, 7.44) and 2.63 (95%CI: 1.41, 4.89) respectively. Compared with the wild-type genotype, the OR for being a non-smoker in carriers of the minor CYP2A6*2 allele was 1.80 (95%CI: 1.24, 2.65). No other significant association was found.

**Table 2:** Genotype frequency distributions (%) in the two study group, i.e. controls (non-smokers) and cases (smokers).

		Non-smokers			Smokers		*P-value for between-group comparison*
	M/M	M/m	m/m	M/M	M/m	m/m	
CYP2A6*2	58.8	27.5	13.7	87.2	12.0	0.8	**<0.001**
CYP2A6*9	89.70	10.3	0.0	87.1	11.3	1.6	0.389
CYP2A6*12	75.0	25.0	0.0	45.4	42.0	12.6	**<0.001**
CYP2A6*1×2	81.3	18.8^a^	-	65.1	34.9^a^	-	0.027
CYP2A13*2	87.5	12.5	0.0	96.7	3.3	0.0	0.017
CYP2A13*3	82.2	15.1	2.7	77.3	22.7	0.0	0.268
CYP2A13*4	98.5	1.5	0.0	97.5	2.5	0.0	0.548
CYP2A13*7	100.0	0.0	0.0	99.2	0.8	0.0	0.652
CYP2B6*4	70.0	25.0	5.0	61.5	25.6	12.8	0.132
CYP2B6*9	50.0	36.0	14.0	56.4	33.3	10.3	0.568
*CHRNA3* 546C>T	70.4	26.8	2.8	56.7	43.3	0.0	0.018
*CHRNA5* 1192G>A	32.3	47.7	20.0	34.4	53.8	11.8	0.316
*5-HTT* LPR	40.9	35.2	23.9	24.0	52.0	24.0	0.029
*HTR2A* -1438A>G	26.8	52.1	21.1	32.8	41.6	25.6	0.364
*OPRM1* 118A>G	74.0	24.6	1.4	67.5	24.7	7.8	0.143
*DRD2-ANKK1* 2137G>A (*Taq1A*)	66.7	22.7	10.6	43.2	42.4	14.4	0.007
*CNR1* 3764C>G	68.5	26.0	5.5	67.5	31.0	1.6	0.257

Abbreviations:

a M, major allele; m, minor allele.

Symbol: ^a^ frequency for M/m or m/m. See text for gene abbreviations. Between-group comparisons of genotype frequencies were corrected for multiple comparisons using the Bonferroni method, in which the threshold *P-*value is obtained by dividing 0.05 by the number of comparisons, i.e. n = 17, corresponding to the 17 polymorphisms we studied (thus, threshold *P-*value = **0.003**). *P-*values below the threshold *P-*value are shown in bold.

### Cohort study: Association between genetic polymorphism and smoking-related phenotypes within the smokers' group

After adjusting for multiple comparisons, the results of the ANOVA test showed a significant genotype effect for the following smoking-related phenotypes (Table 3, all *P<*0.017) (i) mean CPD and CYP2A13*3; (ii) PYS and CYP2A6*2, CYP2A6*1x2, CYP2A13*7, CYP2B6*4 and *DRD2-ANKK1* 2137G>A (*Taq1A*); (iii) nicotine dependence (FTND) and *CYP2A*6*9.

**Table 3:** Association between genotypes and smoking-rrelated phenotypes.

	M/M	M/mCYP2A6*2	m/m	*P*	M/M	M/m*CYP2A*6*9	m/m	*P*	M/M	M/mCYP2A6*12	m/m	*P*	M/M	M/mCYP2A6*1×2	m/m	*P*
**CPD**	25.6±10.0	31.1±11.5	40.0	0.66	26.4±10.6	24.7±8.5	35.0±7.1	0.42	26.6±10.8	27.2±10.7	25.7±8.4	0.87	24.9±10.1	29.1±10.2	-	0.03
**PYS**	37.5±22.8	54.9±26.2	86.0	**0.004**	40.4±25.2	34.6±13.2	36.5±12.0	0.69	39.4±25.3	39.9±22.7	41.5±29.1	0.96	32.8±18.3	53.1±27.9	-	**<0.001**
**FTND**	5.9±2.1	5.3±2.2	3.0	0.21	5.8±2.01	7.1±1.9	2.5±0.7	**0.005**	5.7±2.1	6.0±2.1	5.4±2.5	0.58	6.1±2.1	5.3±1.9	-	0.05
	CYP2A13*2				CYP2A13*3				CYP2A13*4				CYP2A13*7			
**CPD**	26.8±10.5	23.8±7.5	-	0.57	25.3±9.5	31.7±11.9	-	**0.004**	26.8±10.4	20.0±10.0	-	0.20	26.5±10.4	40.0	-	0.20
**PYS**	40.1±24.8	31.8±13.5	-	0.51	38.0±23.3	46.4±28.1	-	0.11	40.5±24.4	13.7±5.1	-	0.06	39.2±23.8	106.0	-	**0.006**
**FTND**	5.8±2.1	7.0±2.3	-	0.25	5.8±2.2	6.0±1.9	-	0.68	5.8±2.1	6.7±2.1	-	0.47	5.8±2.1	8.0	-	0.30
	CYP2B6*4				CYP2B6*9				*CHRNA3* 1192G>A				*CHRNA5* 546C>T			
**CPD**	25.7±10.1	27.5±11.2	28.7±10.6	0.51	26.2±10.0	27.8±10.7	24.8±12.2	0.60	26.5±10.7	26.9±10.1	-	0.80	25.4±13.8	24.8±9.3	30.1±10.2	0.034
**PYS**	34.9±19.8	41.5±26.7	57.5±32.1	**0.004**	35.6±19.6	42.2±26.9	52.1±35.4	0.07	41.3±25.2	37.9±23.7	-	0.40	32.7±17.8	37.8±24.2	45.6±26.4	0.14
**FTND**	5.7±2.2	6.0±1.8	6.4±2.4	0.44	5.8±2.2	6.0±1.9	5.7±2.6	0.90	5.7±2.1	5.9±2.1	-	0.60	5.4±2.3	5.9±2.0	5.9±2.2	0.68

	*5HTT* LPR				*HTR2A* -1438A>G				*OPRM1* 118A>G				*DRD2-ANKK1* 2137G>A (*Taq1A*)			
**CPD**	28.0±10.2	26.2±10.1	25.3±11.1	0.36	28.2±9.5	25.9±11	25.6±10.2	0.52	26.9±9.8	23.0±11.5	32.0±7.9	0.03	27.5±9.4	26.7±10.6	22.3±11.6	0.17
**PYS**	43.7±25.1	38.8±25.8	38.8±19.1	0.63	47.2±27.5	37.4±23.8	37.6±20.9	0.14	40.6±23.6	34.4±23.1	51.4±27.4	0.13	47.9±26.9	35.9±21.1	28.2±14.0	**0.002**
**FTND**	5.9±2.4	5.9±1.8	5.7±2.4	0.93	5.3±1.9	5.9±2.1	6.2±2.2	0.16	5.8±2.1	6.0±2.0	5.4±2.4	0.70	5.7±2.2	5.7±2.1	6.6±1.6	0.278

	*CNR1 3764C>G*			
**CPD**	27.0±10.4	25.4±9.0	27.5±17.7	0.70
**PYS**	38.4±22.77	42.3±26.5	55.0±35.3	0.47
**FTND**	5.9±2.2	5.7±1.7	2±0.0	0.03

Abbreviations: CPD, mean number of cigarettes smoked in one day; FTND, Fagerstrom Test Nicotine dependence test; M. major allele; m. minor allele; NS. non-significant (P>0.05); PYS, mean pack years smoked (see text for explanations). For each polymorphim, between-genotype comparisons were corrected for multiple comparisons using the Bonferroni method, where the threshold *P*-value is obtained by dividing 0.05 by the number of comparisons, i.e. n = 3, corresponding to each genotype (thus, threshold *P*-value = 0.017). *P-*values that were below the threshold *P*-value are shown in bold.

Data are shown as mean±SD values.

## Discussion

Overall, our results suggest that genetic variants that can influence nicotine metabolization (mainly, *CYP2A*6 polymorphisms) are those showing the strongest association with smoking status and smoking-related phenotypes. No significant association was observed for those genetic polymorphisms that are involved in the brain effects of nicotine through nicotinic acetylcholine (*CHRNA3, CHRNA5)*, serotoninergic (*HTR2A*), opioid (*OPRM1*) or cannabinoid receptors (*CNR1*) and serotonin transporters *(5HTT).* The only candidate polymorphism involved in the brain effects of nicotine that was associated with smoking status and with tobacco consumption (expressed as PYS) was *DRD2-ANKK1* 2137G>A (*Taq1A*).

The strongest genetic association we found in our study with smoking status and smoking-related phenotypes was for polymorphisms in *CYP2A6*, the gene encoding the principle nicotine C-oxidase [24]. Smokers who are partially or totally deficient in this enzyme owing to carriage of the variant allele of some *CYP2A6* polymorphisms are ‘poor’ (or ‘slow’) nicotine metabolizers; as such, they are theoretically expected to have a reduced need for cigarette consumption compared with the wild-type genotype [25], [26] However, it is also possible that *prolonged* high levels of brain nicotine owing to reduced metabolization might increase the risk for nicotine dependence, leading to a certain ‘nicotine tolerance’ phenomenon [27], [28]. With regards to these considerations, it must be kept in mind that the decrease in enzyme activity is considerably more marked with carriage of the CYP2A6*2 allele than with the CYP2A6*12 variant. Thus, *2 allele-carriers, who are ‘null-slow’ rather than ‘intermediate metabolizers’ could experience a phenomenon of ‘nicotine tolerance’ with high cigarette consumption (>20 CPD) early in their smoking lifetime [27]. In other words, smokers with the CYP2A6*2 allele might experiment more negative effects when they start to become smokers; but when they continue smoking they may experience prolonged nicotine levels in the brain, thereby becoming more rapidly tolerant and thus needing to smoke more [29]. Our findings are consistent with the aforementioned biological implications of CYP2A6*12 and CYP2A6*2 variants. First, both CYP2A6*2 and CYP2A6*12 polymorphisms were strongly associated with smoking status, yet the variant *2 and *12 alleles were underrepresented and overrepresented respectively in smokers. Second, the CYP2A6*2 variation, but not the CYP2A6*12 polymorphism was associated with smoking phenotypes within the smokers' group, with those individuals homozygous for the *2 allele showing the highest levels of long-term cigarette consumption (PYS). On the other hand, carriage of the CYP2A6*1×2 duplication allele, leading to faster nicotine metabolization was also associated with higher PYS. Our results are in overall agreement with those reported by Rao *et al*, who showed that individuals with the duplication allele CYP2A6*1×2 had higher nicotine consumption [23].

Regarding those genes involved in the central effects of nicotine, we only found a significant association for *DRD2-ANKK1* (*Taq1A*). The variant A1 allele: (i) was associated with an increased chance of being a non-smoker, (ii) tended to be overrepresented in non-smokers compared with smokers (yet the between-group comparison did not withstand statistical correction for multiple comparisons), and (iii) positively associated with PYS in smokers. There is controversy in the literature: previous studies suggested and association of the A1 allele with susceptibility to smoking [30] but more recent studied failed to replicate such association. The *DRD2-ANKK1* gene is involved in the nicotine effects through dopaminergic pathways, with the variant A1 allele being associated with lower density of dopaminergic receptors (DRD2) in the striatum [30]–[32]. Our findings might indeed suggest that people with a functional deficit in the dopamine reward pathway do not experience a reward with smoking initiation, which might confer a protective role to the A1 allele against smoking initiation. However, once they have become smokers, A1-carriers might need to consume more nicotine to enhance the dopaminergic system [30], [33]. This might explain why the A1 allele was positively associated with PYS in our smokers' group.

A novelty of our study stems from the fact that we analyzed the association of the *HTR2A*-1438A>G polymorphism with smoking status and all smoking-related phenotypes, including nicotine dependence. The serotoninergic system could theoretically be implicated in habitual smoking because nicotine increases brain serotonin secretion and nicotine withdrawal has the opposite effect [34], [35]. Polina *et al* found a higher frequency of the variant A allele in European-derived Brazilian smokers than in their non-smoking controls [35]. However, our results do not provide evidence for an association between *HTR2A* -1438A>G and smoking status. Reasons for disparity between the findings reported by Polina *et al* and the present ones might lie, at least partly, in the different ethnic background of the two study cohorts. Notably, the frequency of the A allele was considerably lower in their non-smoking controls (40%) compared with ours (53.6%).

On the other hand, we found no association for those genetic polymorphisms that are involved in the brain effects of nicotine through nicotinic acetylcholine (*CHRNA3, CHRNA5)*, serotoninergic (*HTR2A*), opioid (*OPRM1*), cannabinoid receptors (*CNR1*) or serotonin transporters (*5HTT*). Some studies reported a significant association between the aforementioned variants and nicotine dependence [35]–[39] while others failed to corroborate such association [40]–[43]. A marked racial/ethnic diversity exists in smoking behavior and smoking-related phenotypes (such as age of smoking initiation, smoking rate or level of dependence), as well as in the genotype frequencies of the functional polymorphisms we studied here [44], [45], which could explain, at least partly, differences between studies.

We believe there are several novelties and strengths in our design. This the first association study in the field that takes into account the most important polymorphisms that are strong candidates to influence smoking behavior, i.e. those involved in nicotine metabolization, as well as in the brain effects of nicotine. The results of our study are overall valid, as all the following criteria were met [46]: the studied phenotypes (smoking status and smoking-related phenotypes) were properly defined and accurately recorded by a researcher who was blind to the genetic information; both groups (smokers and non-smokers) were ethnically matched; genotype assessment was unbiased and accurate; we adjusted all statistical inferences for multiple comparisons; and the results are overall consistent with previous research in the field [45], [47]. A weakness of our study was the low sample size of both cohorts, yet we believe this can be partly overcome by the fact that both cohorts were homogeneous and well defined in terms of phenotype assessment.

In conclusion, our results suggest that genetic variants potentially involved in nicotine metabolization (mainly, *CYP2A*6 polymorphisms) are those showing the strongest association with smoking status and smoking-related phenotypes, as opposed to most genetic variants that can influence the brain effects of nicotine, except for the *DRD2-ANKK1* 2137G>A polymorphism. We believe studies as the present ones might help understanding the role of genetics in smoking behavior and on potential smoking cessation, and to better focus therapeutic approaches based on the knowledge of each individual's genetic predisposition to smoking.
